# Could Lipo-Prostaglandin E1 Be the Key to Improving Success Rates in Free-Flap Microsurgery? A Systematic Review

**DOI:** 10.3390/jcm15010092

**Published:** 2025-12-23

**Authors:** Abdullh AlQhtani

**Affiliations:** Plastic Surgery, Department of Surgery, College of Medicine, Prince Sattam bin Abdulaziz University, Al-Kharj 16273, Saudi Arabia; a.alqhtani@psau.edu.sa

**Keywords:** lipo-prostaglandin E1, prostaglandin E1, microsurgery, anticoagulants, vasodilator, reconstruction

## Abstract

**Background**: Microsurgery and free tissue transfer with microanastomoses are common practices that are reliable for restoring anatomical function and/or morphology. Maintaining adequate blood flow to transferred tissue and preventing thrombosis are key challenges in improving the success of surgery. We conducted a systematic review to investigate the use, effects, and efficacy of lipo-prostaglandin E1 (lipo-PGE1) and PGE1, which have vasodilatory and anticoagulation effects, in microsurgery. **Methods**: Studies were reviewed for information about the administration of lipo-PGE1/PGE1, including the purpose, effectiveness, administered doses, and duration of use. This review included articles published up to 2024. Databases: PubMed, MEDLINE, and Embase were searched using the keywords: “flap” AND “prostaglandin E1” and “microsurgery” AND “prostaglandin E1.” **Results**: The initial database search yielded 359 citations; 14 were included in our study with qualitative analysis. These 14 original articles reported PGE1/lipo-PGE1 use in microsurgery for the reconstruction of different anatomical sites, with the most common being the head and neck. Twenty-one different flaps were used; the most common flaps used in head, neck, and lower limb reconstructions were anterolateral thigh flaps. Most studies reported using PGE1/lipo-PGE1 as an antithrombotic, an anticoagulant, a vasodilator, and a strategy to examine blood flow post administration. Only one study compared its effectiveness between two groups and showed significantly lower perfusion-related complications in the prostaglandin group than in the control group. **Conclusions**: Lipo-PGE1/PGE1 has potential vasodilator effects that increase blood flow through free flaps and potential anticoagulant properties that help prevent thrombosis in microanastomoses. However, multicenter, randomized controlled studies are needed to fully elucidate its benefits.

## 1. Introduction

Microsurgery and free tissue transfer with microanastomoses are common procedures that have been shown to be reliable for restoring anatomical function or shape following trauma, tumor resection, or burns and chronic wounds [1]. Maintaining adequate blood flow to the transferred tissue and preventing thrombosis are key challenges in ensuring successful surgery [2].

Many preoperative, intraoperative, and postoperative methods to address these challenges have been described in the literature. Among these studies, various drug interventions have been investigated, with studies reporting the purposes/effects of these drugs, including increasing blood pressure, increasing blood flow, and preventing thrombosis [3].

Prostaglandin is a metabolite of arachidonic acid that can function as an inhibitor of platelet aggregation and as a vasodilator. This product is often used in treating peripheral arterial occlusive disease, Raynaud syndrome, and claudication [4]. Prostaglandin E (PGE1) is a pharmacological agent described as a vasodilator and an inhibitor of platelet aggregation. PGE1 can increase blood flow, prevent thrombosis, and act as a fibrinolytic agent [5,6].

To increase the duration of action and decrease side effects, PGE1 has been modified via encapsulation by using lipid microspheres (carriers) for enhanced stability and targeted delivery. The resulting product is termed lipo-prostaglandin E1 (lipo-PGE1) [7].

Studies have shown that lipo-PGE1/PGE1 can be useful in various medical conditions, including diabetic neuropathy, diabetic foot ulcer, peripheral vascular disease, and acute lower limb ischemia [8,9,10,11]. Recent reports show that lipo-PGE1 use has increased in such procedures as microsurgeries, micro-replantation of the upper limb, perforator-based free flaps, “super-thin flaps,” reconstructive surgeries of the head and neck, oral cavity, and “super” microsurgery [11,12,13,14,15,16]. In addition, lipo-PGE1 has been used as an anticoagulant in microvascular free tissue transfer in reconstructing the lower limb and in routine venous thromboembolism prophylaxis in head and neck reconstructions [17,18].

The aim of this review was to explore the use of lipo-PGE1/PGE1 in microsurgery; the doses, duration, and purpose of use; and its efficacy in free-flap microsurgery in humans.

## 2. Materials and Methods

We conducted a systematic review to explore lipo-PGE1/PGE1 use in free-flap microsurgery in humans and specifically investigate the purpose, efficacy, administered doses, and duration of use. This review was conducted using the Preferred Reporting Items for Systematic Reviews and Meta-Analyses (PRISMA) guidelines, and the PRISMA checklist was included as Supplementary Materials. Our review was conducted according to International Prospective Register of Systematic Reviews (PROSPERO) guidelines with the ID number CRD420251236403.

### 2.1. Information Sources and Search Strategy

A systematic search of electronic databases (PubMed, MEDLINE, and Embase) was conducted. In addition, a manual search of the reference lists of the identified articles was conducted to determine their relevance.

The author and two independent reviewers conducted the comprehensive search using the following keywords: “flap” AND “prostaglandin E1” and “microsurgery” AND “prostaglandin E1” “flap” AND “lipo-prostaglandin E1” and “microsurgery” AND “lipo-prostaglandin E1” and “flap” AND “Alprostadil” and “microsurgery” AND “Alprostadil.” The studies collected by the author and two independent reviewers are listed.

### 2.2. Inclusion and Exclusion Criteria

The primary aim of this investigation was to identify all studies reporting free-flap microsurgery using lipo-PGE1/PGE1 and determine its purpose and efficacy. We excluded non-human studies, case reports, systematic reviews, editorials, author response theses, and conference reports. Non-English, duplicated, unrelated, abstract-only articles, and unavailable full texts were also excluded.

### 2.3. Study Endpoints

The primary endpoint of this study was the use of lipo-PGE1/PGE1 in free-flap microsurgery. The secondary endpoints were purpose, efficacy, dosage, and duration of use.

### 2.4. Data Abstraction, Study Selection, and Quality Process

We searched for studies published up to December 2024. Studies were imported into Endnote 21 and subsequently screened to eliminate unrelated studies, systematic reviews, and duplicates. Thereafter, all full-text articles were screened; study designs and inclusion criteria were systematically documented. Disagreements were resolved by a third reviewer. The risk of bias for each included study was evaluated using the ROBINS-I tool. This evaluation was also performed by the two independent reviewers to ensure the assessment was consistent and reliable.

## 3. Results

### 3.1. Characteristics of Included Studies

**Figure 1:**
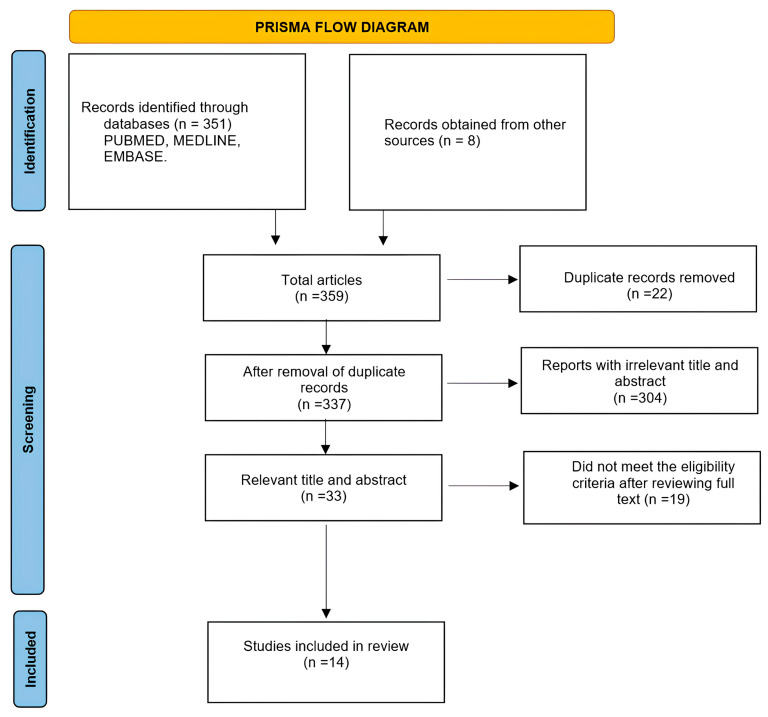
Preferred Reporting Items for Systematic Reviews and Meta-Analyses (PRISMA) flow diagram.

The initial search yielded 359 citations from the different databases. After removal of duplicates, 337 remaining articles were fit for full-text screening (Figure 1). The titles and abstracts of the remaining studies were reviewed, yielding 33 relevant abstracts. After full-text evaluation, 19 studies did not meet the inclusion criteria. Ultimately, 14 articles were included in this full-text review (Table 1).

### 3.2. Risk of Bias Assessment

By using the ROBINS-I tool, 14 studies were assessed. Most of the concerns were about the selection of participants. Overall, most of the studies showed a low to moderate risk of bias, as shown in Table 2.

### 3.3. Types of Surgery

The most common sites of reconstruction were the head and neck; there were four studies on the head and neck, one on oral cavities, and one on the temple area. Lower limb reconstruction was mentioned in three studies, two on the lower limb in general and one on the knee area. One study included micro-replantations in the hand after traumatic amputations.

The remaining five studies included descriptions of different locations of reconstructions in single papers, which collectively included the head and neck, breast, trunk, upper limb, and lower limb.

A total of 21 flaps were used in the studies. The most common flaps used in head and neck reconstructions were anterolateral thigh, jejunum, radial forearm, and rectus abdominis musculocutaneous flaps. Anterolateral thigh flaps were most commonly used in lower limb reconstructions (Table 3).

### 3.4. Medications

The relevant medications were mentioned in the studies under different names, including Alprostadil, prostaglandin E1, and lipo-prostaglandin E1. Furthermore, trade names were used in five studies, namely, Eglandin in four and Promostan in one. The dosage was slightly different between studies; however, the range was between 10 and 80 mcg per day, and most of the studies reported using the relevant medications for 5–7 days. Additionally, most studies reported using these medications postoperatively, except for one in which the first dose was administered intraoperatively. Almost all the studies reported that the medications were administered intravenously, except for one study, in which medications were administered via catheter into the femoral artery proximal to the anastomosed site in a lower limb reconstruction (Table 4).

### 3.5. Uses

Two of the fourteen studies reported results from their early, i.e., preliminary, experience in using prostaglandins. Rodríguez Vegas et al. used prostaglandins to prevent and treat spasms and thrombosis during microsurgery [12]. After tumor resection from the lower limb and microsurgery reconstruction, Saito et al. inserted prostaglandins via a catheter into the femoral artery proximal to the anastomosis as a standard continuous infusion, with the addition of heparin as an anticoagulant [17].

Five studies reported using prostaglandin as an antithrombotic or anticoagulant. Owing to the high risk of hemorrhagic complications, superior effects of PGE1 on anastomosis, and prolonged duration of these effects, Fukuiwa et al. routinely used PGE1 as an antithrombotic drug [19]. Yoshimoto et al. routinely administered PGE1 with heparin to prevent deep vein thrombosis after head and neck reconstruction surgeries [13]. Riva et al. compared a group in which dextran-40 was used as an antithrombotic in free tissue transfer for head and neck surgeries with a control group (no antithrombotic) [20]. Lin et al. used dextran-40 and PGE1 as postoperative anticoagulants for microsurgery tissue transfer in hematological disease [21]. Wang et al. studied risk factors for flap failure in the head and neck and used PGE1 as an anticoagulant; however, it was used in only a few patients [23]. Two of the fourteen studies reported routinely using vasodilators. Hong et al. routinely used PGE1 as a vasodilator and perforators as the recipient vessels [14]. Goh et al. routinely used a superficial circumflex iliac artery perforator flap as an ideal thin skin flap and PGE1 as a vasodilator [16]. Jung et al. reconstructed the temple area with a lateral arm free flap and routinely used PGE1; these authors did not state their rationale for using PGE1 [25]. Park & Lee (2024) retrospectively studied the clinical effects of PGE1 in reducing flap necrosis by comparing a group of patients who received PGE1 with a control group [26].

Three of the fourteen studies examined the effect of PGE1 on blood flow in the flap post anastomosis. Mitate et al. found that average blood flow rates increased gradually until reaching a peak on postoperative day 7; however, after PGE1 injection, blood flow reached a peak between 75 and 105 min [15]. Using duplex ultrasonography, Jin et al. tested the arterial inflow before and after administering lipo-PGE1 and found that the arterial inflow increased 30 min after administration [22]. In contrast, Park et al. [24] (2020) did not find significant differences in arterial inflow before and 30 min after lipo-PGE1 infusion in patients with diabetes who underwent lower limb reconstruction, except in those aged <65 years (Table 5).

### 3.6. Effectiveness and Complications

There are many definitions of flap failure among the included studies; it is defined as complete flap loss in some studies and as partial flap necrosis or perfusion-related complications requiring surgical intervention in others. We tried to distinguish between total flap loss and partial complications in our data presentation as much as possible. Rodríguez Vegas et al. reported 16 micro-replantations in the hand and 46 free flaps in different locations without encountering flap loss or an increased incidence of complications. However, their study used multiple medications, including anticoagulants such as aspirin and heparin, in addition to PGE1 [12]. Saito et al. studied 11 cases of microvascular reconstruction in the lower limbs using PGE1 and heparin as anticoagulants for continuous infusion and reported no flap loss or serious systemic complications [17]. Fukuiwa et al. reviewed 102 cases of head and neck reconstruction with a free flap to investigate the incidence of venous thrombosis when PGE1 was used as an antithrombotic drug and reported six cases of venous thrombosis due to recipient vessels such as the external and internal jugular veins [19].

Yoshimoto et al. conducted a large retrospective study of 59 cases of postoperative thrombosis in 1031 patients who underwent free-flap reconstruction after head and neck cancer surgery and were routinely administered PGE1 as an antithrombotic. Those cases with flap salvage had a 4.8% flap loss with a high success rate of reanastomosis for the radial forearm compared with cases using jejunal flaps [13]. Riva et al. compared a dextran-40 group, PGE1 (as an antithrombotic) group, and a control group in 1351 patients who underwent reconstructive microsurgery of the head and neck. The authors found no significant differences between the PGE1, dextran-40, and control groups, except for a significant increase in the flap failure rate in patients with comorbidities who were administered dextran-40 [20]. Lin et al. reviewed 26 free flaps in 20 patients with hematological disorders using dextran-40 and PGE1 as anticoagulant medications; their study reported three flap failures [21].

Wang et al. conducted a large retrospective study including 21,548 patients who underwent head and neck reconstruction using multiple anticoagulant medications; PGE1 was only used in eight patients, and the total flap failure rate in the study was 4.1% [23]. Hong et al. conducted a prospective study of 25 free flaps using perforators as recipient vessels to reconstruct the knee area using PGE1 as a vasodilator and reported no complications or flap loss [14]. Goh et al. reviewed 210 cases of reconstructive surgery using superficial circumflex iliac artery perforator flaps as thin flaps and PGE1 as a vasodilator, finding a 4.8% total flap loss [16]. Jung et al. conducted a retrospective study of 12 patients in whom lateral arm free flaps were used to reconstruct the temple area. The surgeons routinely used PGE1 and reported no complications or flap loss [25]. Park et al. [24] conducted a comparative retrospective study on the clinical effectiveness of PGE1 in 274 free flaps: 142 received PGE1, and 132 did not. The PGE1 group showed significantly lower perfusion-related complications than the non-PGE1 group. The total flap loss was 2.1% in the PGE1 group versus 2.3% in the non-PGE1 group; this difference was not statistically significant (Table 5) [24].

## 4. Discussion

Improving microsurgery outcomes remains a challenge that can be overcome by ensuring adequate blood flow to transferred tissue and preventing thrombosis.

Antithrombotic medications are used routinely; however, few studies have shown their efficacy in microsurgery in humans or fully elucidated and compared their side effects. Animal studies have shown the efficacy of prophylactic anticoagulation [27,28]. The optimal medications that should be administered for thrombosis remain unelucidated and depend on the different causes and explanations [29,30].

Aspirin helps prevent platelet aggregation; however, it carries a risk of gastric ulceration and nephrotoxicity. Heparin prevents fibrin aggregation, which can induce thrombocytopenia. Dextran prevents platelet and fibrin aggregation but has a high risk of systemic complications [28,31].

PGE1 was used as an anticoagulant and antithrombotic medication in many studies in our review; however, these were not randomized controlled studies. In addition, the PGE1 sometimes used in these studies was concomitant with other anticoagulants.

Riva et al. performed a retrospective review of the outcomes of three groups of patients who received dextran-40, PGE1, and no antithrombotic medications, and found no significant differences in flap survival. However, their study was retrospective, and the medications were administered according to surgeons’ preferences [20].

Lin et al. reviewed the outcomes of patients with hematological disease who underwent flap transfer using the anticoagulant heparin with dextran-40 or heparin with PGE1 and reported an overall success rate of 88.5% [21].

Only one study compared the use of PGE1 with non-PGE1 in patients undergoing microsurgery; Park and Lee (2024) conducted a retrospective study on the clinical effectiveness of PGE1 that showed reduced risks of partial flap necrosis development [26].

Vasodilators used in microsurgery help prevent vasospasm, which can jeopardize blood flow, leading to stasis and anastomotic clotting. Many agents described in the literature are used locally during anastomosis, such as phosphodiesterase inhibitors, local anesthetics, calcium channel blockers, alpha antagonists, and direct vasodilators [32]. These are local agents; therefore, their effects are limited during surgery, which raises the question of whether a vasodilator exists that has a longer duration of action with fewer systemic side effects. Jin et al. showed that continuous infusion of 0.4 μg/h of lipo-PGE1 is safe for patients undergoing microsurgery; the hemodynamic status of the patients before and after lipo-PGE1 administration did not significantly differ [22]. The differences in the reported effects of lipo-PGE1/PGE1 on hemodynamics may be due to differences in infusion rates and patient characteristics.

Two studies showed that PGE1 administration resulted in significantly increased blood flow velocity 30 min after administration and reached a peak between 75 and 105 min postoperatively instead of at day 7 sans PGE1 [15,22].

PGE1 may help indirectly link vessel dilation, which includes dilation of the arterioles and choke vessels that allow for the capture of more perforasomes. This process can be limited in patients over 65 years of age with diabetes owing to the pathophysiology of diabetic vascular disease. Park et al. (2020) recommended using PGE1 for 3 days to achieve effective dilatation of the microcirculation and conducted further research to determine the optimal time for PGE1 usage [24].

The flap failure rate for head and neck reconstruction varies from 0.8% to 10.6%; our literature search of articles reporting the use of lipo-PGE1/PGE1 revealed a failure rate between 0% and 6% [33,34].

According to Serra et al., the total flap necrosis rate in lower limb reconstruction was 7.78%, most often occurring in patients who sustained trauma [35]. Our review showed a failure rate between 0% and 7.5%, specifically in patients with diabetes who are considered a more challenging population. Moreover, we found that 7.5% of the patients with diabetes who have undergone lower limb reconstruction ultimately require amputation.

We hypothesize that using lipo-PGE1/PGE1 for at least 3 days increases blood flow in the transferred tissue at a certain dosage via a vasodilatory effect. In addition, lipo-PGE1/PGE1 has an anticoagulation effect that may help decrease thrombotic events in microanastomosis and deep venous thrombosis in lengthy procedures; however, further studies are needed to reveal the definitive benefits of its use.

Our findings suggest that there is a potential benefit of PGE1 in microsurgery, but it is important to know that there are many alternative approaches. Riccardo et al. recently reported a 95.7% overall success rate in head and neck free-flap reconstruction without using PGE1 [36].

Multiple variables were reported in the studies included in this review. The quality of the methodology of the included studies must be considered while interpreting our findings because around half of the studies included had a serious risk of bias due to the selection of participants, and most of the studies were retrospective or non-randomized. Also, the use of other anticoagulants along with lipo-PGE1/PGE1 may have affected the results, although there are studies that used PGE1/PGE1 without combination with other medication. Other limitations included the lack of a control group, differences in doses and patients’ comorbidities, and retrospective studies that insufficiently explored alternatives to PGE1. Therefore, a comprehensive meta-analysis was not possible, and there is a need for well-designed, multicenter, randomized controlled trials to validate these findings and establish evidence-based guidelines for the optimal use of lipo-PGE1/PGE1 in microsurgical reconstruction.

## 5. Conclusions

Our review showed that PGE1/lipo-PGE1 has potential vasodilator effects that increase blood flow in free-flap reconstruction surgeries and the potential anticoagulant effects that help prevent thrombosis in microanastomoses; however, multicenter, randomized controlled studies are needed to elucidate its benefits.

## Figures and Tables

**Table 1:** Summary of studies.

Study	Type of Study	Surgery	Limitations of the Study
Rodríguez Vegas, Ruiz Alonso & Terán Saavedra (2007) [12]	retrospective	16 micro-replantations 46 free flaps (different locations)	No control group + other medications
Fukuiwa 2008 [19]	retrospective	102 free flaps (head and neck)	No control group
Yoshimoto 2010 [13]	retrospective	1031 free flaps (head and neck)	No control group + other medications
Saito et al. (2010) [17]	case series	11 free flaps (lower limb)	No control group + other medications
Hong & Koshima (2010) [14]	prospective	25 free flaps using perforators as recipient vessels. (knee joint area)	No control group + other medications
Mitate et al. (2011) [15]	prospective	14 free microvascular flaps (oral cavity)	No control group + dose not mentioned
Riva et al. (2012) [20]	retrospective	1351 free flaps 232 PGE1 283 dextran 836 no antithrombotic (head and neck)	No exclusion criteria
Lin et al. (2014) [21]	retrospective	26 free flaps in 20 patients with hematological disorders (different locations)	No control group + other medications + hematologic disorder
Goh 2015 [16]	retrospective	210 superficial circumflex iliac artery perforator flaps (different locations)	No control group + other medications
Jin 2019 [22]	prospective	37 patients undergoing free-flap reconstruction. Superficial circumflex iliac artery perforator flap and anterolateral thigh flap (different locations)	No control group
Wang et al. (2020) [23]	retrospective	21,548 patients, only 8(0.04%) received PGE1 (head and neck)	No control group + other medications + the results, implications, and applicability should be evaluated
Park et al. (2020) [24]	prospective observational study	40 patients with diabetes with free flap (lower limb)	No control group + other medications + multiple factors
Jung, Lee & Eun (2024) [25]	retrospective	12 patients’ reconstructions with lateral arm free flaps (temple area)	No control group
Park & Lee (2024) [26]	retrospective	274 free flaps 142 PGE1 132 no PGE1 (different locations)	Rates of comorbidities and chronic related wounds were higher in the PGE1 cohort in the baseline characteristics

**Table 2:** ROBINS-I assessment of non-randomized trials.

Study	D1	D2	D3	D4	D5	D6	D7	Overall
Rodríguez Vegas, Ruiz Alonso & Terán Saavedra (2007) [12]	Moderate	Moderate	Low	Low	Low	Low	Low	Moderate
Fukuiwa 2008 [19]	Moderate	Moderate	Low	Low	Low	Low	Low	Moderate
Yoshimoto 2010 [13]	Moderate	Moderate	Low	Low	Low	Low	Low	Moderate
Saito et al. (2010) [17]	Moderate	Serious	Low	Low	Low	Low	Low	Moderate
Hong & Koshima (2010) [14]	Moderate	Serious	Low	Low	Low	Low	Low	Moderate
Mitate et al. (2011) [15]	Moderate	Serious	Low	Low	Low	Low	Low	Moderate
Riva et al. (2012) [20]	Moderate	Serious	Low	Low	Low	Low	Low	Moderate
Lin et al. (2014) [21]	Moderate	Serious	Low	Low	Low	Low	Low	Moderate
Goh 2015 [16]	Moderate	Moderate	Low	Low	Low	Low	Low	Moderate
Jin 2019 [22]	Moderate	Serious	Low	Low	Moderate	Low	Low	Moderate
Wang et al. (2020) [23]	Low	Moderate	Low	Low	Moderate	Low	Low	Low
Park et al. (2020) [24]	Low	Moderate	Low	Low	Low	Low	Low	Low
Jung, Lee & Eun (2024) [25]	Moderate	Serious	Low	Low	Low	Low	Low	Moderate
Park & Lee (2024) [26]	Low	Moderate	Low	Low	Moderate	Low	Low	Low

**Table 3:** Summary of locations of surgeries and flaps.

Area of Reconstruction	No. of Studies	Flaps Used
Head and neck	4	Jejunum, RF, RA LD, ALT, FF, others
Oral cavity	1	RF, RA, LD
Temple area	1	LA
Lower limb	2	LL, LD, SF, ALT, TFL, SCIP, PAP, DPA
Knee area	1	ALT, MTP
Upper limb	1	Micro-replantations
Different locations *(head and neck, breast, trunk, upper limb and lower limb)*	5	ALT, DIEP, SIEA, TAP, FF, RA, RF, GF, SA, SCIP, VL, TDAP, LD, RASP

ALT: anterolateral thigh; DIEP: deep inferior epigastric perforator; SIEA: superficial inferior epigastric artery; TAP: thoracodorsal artery perforator; RF: radial forearm; RA: rectus abdominis; LD: latissimus dorsi; SA: serratus anterior; TFL: tensor fascia lata; FF: fibula free; GF: groin flap; SCIP: superficial circumflex iliac artery perforator; PAP: profunda artery perforator; DPA: dorsalis pedis artery; VL: vastus lateralis; TDAP: thoracodorsal artery perforator; RASP: radial artery superficial palmar; FA: forearm; LA: lateral arm; SF: scapular flap; MTP: medial thigh perforator.

**Table 4:** Summary of drug names, administration, dose, and time.

	Name of Drug	Drug Administration	Dose and Days	Time of Injection
Rodriguez Vegas 2007 [12]	Alprostadil	- Intravenous - Intravenous	- 40 mcg every 12 h - 40 mcg every 12 h 5–7 days	- Intraoperative - Postoperative
Fukuiwa 2008 [19]	Alprostadil	Intravenous	80 mcg for 5 days	Postoperative
Yoshimoto 2010 [13]	PGE1	Intravenous	80 mcg/day for 5 days	Postoperative
Saito et al. (2010) [17]	PGE1	Continuous infusion in the limb	40 mcg for 7 days	Postoperative
Hong & Koshima (2010) [14]	Lipo-PGE1 Eglandin	Intravenous	10 mcg + 5% DW for 5 days	Postoperative
Mitate et al. (2011) [15]	PGE1	Intravenous	Twice daily for 7 days	Postoperative
Riva et al. (2012) [20]	PGE1, PROMOSTAN	Intravenous	80 mcg for 5–7 days	Postoperative
Lin et al. (2014) [21]	Prostaglandin-E1	Intravenous (heparin), postoperative (dextran-40 or PGE1)	Not specified	Postoperative
Goh 2015 [16]	Prostaglandin-E1	Intravenous	10 mcg + 5% DW for 5 days	Postoperative
Jin 2019 [22]	Lipo-prostaglandin E1	Intravenous	Rate of 0.4 μg/h	Postoperative
Wang et al. (2020) [23]	Prostaglandin (PGE1),	Not specified	Not specified	Not specified
Park et al. (2020) [24]	Lipo-prostaglandin E1	Intravenous	Rate of 0.4 μg/h	Postoperative
Jung et al. (2024) [25]	Alprostadil (PGE1, Eglandin^®^)	Intravenous	Not specified	Postoperative
Park & Lee (2024) [26]	PGE1, Eglandin^®^	Intravenous	10 mcg + 100 cc normal saline over 2 h for 7 days	Postoperative

PGE1, prostaglandin E1; lipo-PGE1, lipo-prostaglandin E1; DW, dextrose in water.

**Table 5:** Summary of usage and flap failure.

	Routine or Examination Use	% of Flap Failure
Rodríguez Vegas et al. (2007) [12]	Preliminary/early experience using prostaglandins as anticoagulants	0%
Fukuiwa et al. (2008) [19]	Routine use as antithrombotic drug	5.9%
Yoshimoto 2010 [13]	Routine use as antithrombotic drug	4.8%
Saito et al. (2010) [17]	Preliminary/early experience using prostaglandins anticoagulants via continuous infusion	0%
Hong & Koshima (2010) [14]	Routine use as vasodilator	0%
Mitate et al. (2011) [15]	To determine postoperative pattern of blood flow and reveal effects of PGE1	0%
Riva et al. (2012) [20]	Prostaglandin E1 (PGE1) group in which PGE1 was used as an antithrombotic and compared with dextran-40 and control groups	6% PGE1 6% dextran 5% no antithrombotic therapy
Lin et al. (2014) [21]	Routine use as anticoagulant with dextran	11.5% (3/26)
Goh 2015 [16]	Routine	4.8%
Jin 2019 [22]	Use of multiple anticoagulants; PGE1 only used in eight patients	Not specified
Wang et al. (2020) [23]	To identify risk factors associated with free-flap failure	4.1%
Park et al. (2020) [24]	To elucidate the role of age, hemoglobin A1c (HbA1c), duration of diabetes, and flap type in determining the effects of lipo-PGE1 infusion on immediate arterial maximal flow velocity	7.5% required amputation
Jung et al. (2024) [25]	Routine	0%
Park & Lee [16,26]	To identify clinical effectiveness of PGE1 in reducing flap necrosis	Total loss (2.1% vs. 2.3%) Flap: any loss (total, partial, or tip) 7% vs. 18.2%

## Data Availability

The data are available from the corresponding author upon reasonable request.

## Supplementary Materials

The following supporting information can be downloaded at: https://www.mdpi.com/article/10.3390/jcm15010092/s1, PRISMA_2020_abstract_checklist [37].

## Abbreviations

The following abbreviations are used in this manuscript:

PGE1	Prostaglandin E1
