# Supplementary material for: Could Lipo-Prostaglandin E1 Be the Key to Improving Success Rates in Free-Flap Microsurgery? A Systematic Review
Source: J Clin Med. 2025 Dec 23;15(1):92. doi: 10.3390/jcm15010092 (PMC12786470; doi:10.3390/jcm15010092)
Supplement: Supplementary file 1 [file jcm-15-00092-s001.zip › jcm-4037918-supplementary.pdf]

PRISMA_2020_abstract_checklist

Section and Topic	Item #	Checklist item	Reported (Yes/No)
**TITLE**			
Title	1	Identify the report as a systematic review.	YES
**BACKGROUND**			
Objectives	2	Provide an explicit statement of the main objective(s) or question(s) the review addresses.	YES
**METHODS**			
Eligibility criteria	3	Specify the inclusion and exclusion criteria for the review.	YES
Information sources	4	Specify the information sources (e.g. databases, registers) used to identify studies and the date when each was last searched.	YES
Risk of bias	5	Specify the methods used to assess risk of bias in the included studies.	NO
Synthesis of results	6	Specify the methods used to present and synthesise results.	YES
**RESULTS**			
Included studies	7	Give the total number of included studies and participants and summarise relevant characteristics of studies.	YES
Synthesis of results	8	Present results for main outcomes, preferably indicating the number of included studies and participants for each. If meta-analysis was done, report the summary estimate and confidence/credible interval. If comparing groups, indicate the direction of the effect (i.e. which group is favoured).	YES
**DISCUSSION**			
Limitations of evidence	9	Provide a brief summary of the limitations of the evidence included in the review (e.g. study risk of bias, inconsistency and imprecision).	YES
Interpretation	10	Provide a general interpretation of the results and important implications.	YES
**OTHER**			
Funding	11	Specify the primary source of funding for the review.	YES
Registration	12	Provide the register name and registration number.	YES

From: Page MJ, McKenzie JE, Bossuyt PM, Boutron I, Hoffmann TC, Mulrow CD, et al. The PRISMA 2020 statement: an updated guideline for reporting

systematic reviews. BMJ 2021;372:n71. doi: 10.1136/bmj.n71. This work is licensed under CC BY 4.0. To view a copy of this license, visit https://creativecommons.org/licenses/by/4.0/

PRISMA_2020_checklist

Section and Topic	Item #	Checklist item	Location where item is reported
**TITLE**			
Title	1	Identify the report as a systematic review.	Title
**ABSTRACT**			
Abstract	2	See the PRISMA 2020 for Abstracts checklist.	In the attached file in the email
**INTRODUCTION**			
Rationale	3	Describe the rationale for the review in the context of existing knowledge.	Lines 35-60
Objectives	4	Provide an explicit statement of the objective(s) or question(s) the review addresses.	Lines 61-62
**METHODS**			
Eligibility criteria	5	Specify the inclusion and exclusion criteria for the review and how studies were grouped for the syntheses.	Section 2,2 Lines 79-84
Information sources	6	Specify all databases, registers, websites, organisations, reference lists and other sources searched or consulted to identify studies. Specify the date when each source was last searched or consulted.	Section 2,1<br>Lines 71-78
Search strategy	7	Present the full search strategies for all databases, registers and websites, including any filters and limits used.	Section 2,1<br>Lines 71-78
Selection process	8	Specify the methods used to decide whether a study met the inclusion criteria of the review, including how many reviewers screened each record and each report retrieved, whether they worked independently, and if applicable, details of automation tools used in the process.	Section 2,1<br>Lines 71-78
Data collection process	9	Specify the methods used to collect data from reports, including how many reviewers collected data from each report, whether they worked independently, any processes for obtaining or confirming data from study investigators, and if applicable, details of automation tools used in the process.	Section 2.4 lines 88-93
Data items	10a	List and define all outcomes for which data were sought. Specify whether all results that were compatible with each outcome domain in each study were sought (e.g. for all measures, time points, analyses), and if not, the methods used to decide which results to collect.	Section 2.3 lines 85-87
	10b	List and define all other variables for which data were sought (e.g. participant and intervention characteristics, funding sources). Describe any assumptions made about any missing or unclear information.	Section 2.3 lines 85-87
Study risk of bias	11	Specify the methods used to assess risk of bias in the included studies, including details of the tool(s) used, how many reviewers	2 independent reviewer + author.

Section and Topic	Item #	Checklist item	Location where item is reported
assessment		assessed each study and whether they worked independently, and if applicable, details of automation tools used in the process.	Mentioned in 2.1
Effect measures	12	Specify for each outcome the effect measure(s) (e.g. risk ratio, mean difference) used in the synthesis or presentation of results.	NOT APPLICABLE. No meta-analysis performed; narrative synthesis only (justified in Discussion, line 77).
Synthesis methods	13a	Describe the processes used to decide which studies were eligible for each synthesis (e.g. tabulating the study intervention characteristics and comparing against the planned groups for each synthesis (item #5)).	Sections 2.2 and Results. Inclusion/exclusion criteria provided.
	13b	Describe any methods required to prepare the data for presentation or synthesis, such as handling of missing summary statistics, or data conversions.	Section 2.4
	13c	Describe any methods used to tabulate or visually display results of individual studies and syntheses.	Results section. Tables 1-4 present study characteristics, locations, medications, and usage.
	13d	Describe any methods used to synthesize results and provide a rationale for the choice(s). If meta-analysis was performed, describe the model(s), method(s) to identify the presence and extent of statistical heterogeneity, and software package(s) used.	In the Discussion States meta-analysis not possible due to heterogeneity
	13e	Describe any methods used to explore possible causes of heterogeneity among study results (e.g. subgroup analysis, meta-regression).	Int the Discussion Mentions multiple variables causing heterogeneity
	13f	Describe any sensitivity analyses conducted to assess robustness of the synthesized results.	NOT APPLICABLE. No sensitivity analyses conducted (meta-analysis not performed).
Reporting bias	14	Describe any methods used to assess risk of bias due to missing results in a synthesis (arising from reporting biases).	not conducted

Section and Topic	Item #	Checklist item	Location where item is reported
assessment			because this descriptive systematic review aimed to document current practices rather than compare interventions, the overwhelming majority of included studies (13 of 14) lacked control groups, substantial heterogeneity precluded meta-analysis, and study limitations
Certainty assessment	15	Describe any methods used to assess certainty (or confidence) in the body of evidence for an outcome.	Not applicable
**RESULTS**			
Study selection	16a	Describe the results of the search and selection process, from the number of records identified in the search to the number of studies included in the review, ideally using a flow diagram.	Section 3.1 and Figure 1. PRISMA flow diagram showing 359 citations → 22 duplicates removed → 337 screened → 33 relevant → 19 excluded → 14 included.
	16b	Cite studies that might appear to meet the inclusion criteria, but which were excluded, and explain why they were excluded.	All studies were included
Study characteristics	17	Cite each included study and present its characteristics.	Table 1 and throughout Results. All 14 included studies cited with characteristics.

Section and Topic	Item #	Checklist item	Location where item is reported
Risk of bias in studies	18	Present assessments of risk of bias for each included study.	not conducted
Results of individual studies	19	For all outcomes, present, for each study: (a) summary statistics for each group (where appropriate) and (b) an effect estimate and its precision (e.g. confidence/credible interval), ideally using structured tables or plots.	Tables 1-4 and Results. Descriptive data presented
Results of syntheses	20a	For each synthesis, briefly summarise the characteristics and risk of bias among contributing studies.	Tables and Results. Study characteristics summarized.
	20b	Present results of all statistical syntheses conducted. If meta-analysis was done, present for each the summary estimate and its precision (e.g. confidence/credible interval) and measures of statistical heterogeneity. If comparing groups, describe the direction of the effect.	NOT APPLICABLE. No meta-analysis performed (narrative synthesis only).
	20c	Present results of all investigations of possible causes of heterogeneity among study results.	NOT APPLICABLE. No formal heterogeneity analysis.
	20d	Present results of all sensitivity analyses conducted to assess the robustness of the synthesized results.	NOT APPLICABLE. No sensitivity analyses conducted.
Reporting biases	21	Present assessments of risk of bias due to missing results (arising from reporting biases) for each synthesis assessed.	not conducted
Certainty of evidence	22	Present assessments of certainty (or confidence) in the body of evidence for each outcome assessed.	not conducted
**DISCUSSION**			
Discussion	23a	Provide a general interpretation of the results in the context of other evidence.	Discussion section Findings discussed in context of existing literature on antithrombotic medications and vasodilators.
	23b	Discuss any limitations of the evidence included in the review.	Discussion Limitations discussed:

Section and Topic	Item #	Checklist item	Location where item is reported
			heterogeneity, lack of control groups, retrospective nature, variable dosing.
	23c	Discuss any limitations of the review processes used.	Discussion States meta-analysis not possible.
	23d	Discuss implications of the results for practice, policy, and future research.	Discussion and Conclusion (lines 76-80). Recommends multicenter RCTs; suggests further research on optimal duration and dosing.
**OTHER INFORMATION**			
Registration and protocol	24a	Provide registration information for the review, including register name and registration number, or state that the review was not registered.	Methods section PROSPERO registration ID: CRD420251236403
	24b	Indicate where the review protocol can be accessed, or state that a protocol was not prepared.	Methods section PROSPERO ID provided
	24c	Describe and explain any amendments to information provided at registration or in the protocol.	Not mentioned because the is not
Support	25	Describe sources of financial or non-financial support for the review, and the role of the funders or sponsors in the review.	Author declares no funding after the Conclusions section
Competing interests	26	Declare any competing interests of review authors.	Author declares no conflicts of interest after the Conclusions section

Section and Topic	Item #	Checklist item	Location where item is reported
Availability of data, code and other materials	27	Report which of the following are publicly available and where they can be found: template data collection forms; data extracted from included studies; data used for all analyses; analytic code; any other materials used in the review.	Data available from corresponding author upon request after the Conclusions section

From: Page MJ, McKenzie JE, Bossuyt PM, Boutron I, Hoffmann TC, Mulrow CD, et al. The PRISMA 2020 statement: an updated guideline for reporting systematic reviews. BMJ 2021;372:n71. doi: 10.1136/bmj.n71. This work is licensed under CC BY 4.0. To view a copy of this license, visit https://creativecommons.org/licenses/by/4.0/
